# Mosaicplasty of the Femoral Head: A Systematic Review and Meta-Analysis of the Current Literature

**DOI:** 10.7759/cureus.31874

**Published:** 2022-11-24

**Authors:** Vasileios Athanasiou, Evangelia Argyropoulou, Panagiotis Antzoulas, John Lakoumentas, George Diamantakis, John Gliatis

**Affiliations:** 1 Department of Orthopaedics and Traumatology, University General Hospital of Patra, Patras, GRC; 2 Department of Medical Physics, School of Medicine, University of Patras, Patras, GRC

**Keywords:** young adult hip, osteochondral defect, joint-preserving surgery, surgical hip repair, mosaicplasty

## Abstract

Osteochondral lesions of the femoral head are rare. For the treatment of these lesions, various joint-preserving procedures, particularly in young, active patients, have been developed. Mosaicplasty is a well-established surgical procedure for the knee. However, there is little evidence that this method can also be used to treat osteochondral lesions in the hip. The indication for cartilage procedures continues to evolve for the knee, and a similar strategy may be adopted for the hip joint. Due to limited evidence and a lack of experience, mosaicplasty treatment of these lesions remains challenging, especially in young patients. This study shows that open and arthroscopic management using the knee and femoral head as donor sites yielded good to excellent short- to mid-term outcomes. For osteochondral lesions of the femoral head, mosaicplasty may be a new alternative treatment option, although this needs to be proven with longer follow-ups and in a larger sample of patients.

## Introduction and background

The most common cause of mechanical symptoms in the hip is labral tears and cartilage lesions [1,2]. Neumann et al. found that up to 76% of patients (from the age of 17 to 76 years old) presenting with mechanical hip complaints have hip chondral lesions visible on magnetic resonance imaging (MRI) [1]. Trauma, labral tears, femoroacetabular impingement (FAI), arthritis, osteonecrosis, and dysplasia have been identified as causative factors [1-4]. Osteochondral lesions of the femoral head account for only about 2% of all osteochondral lesions. A study revealed that the frequency might be as high as 18% in asymptomatic professional hockey players [5]. Cartilage injury of the hip is a risk factor that can lead to progressive joint degeneration and severe disability, especially in young patients due to cartilage’s poor regeneration capabilities. Magnetic resonance arthrography (MRA), arthroscopy, and non-contrast 3-Tesla magnetic resonance imaging (3-T MRI) are useful tools for the assessment of internal pathology of the hip [6]. On the arthroscopic evaluation of 457 hips, McCarthy and Lee found that most chondral injuries (59%) were associated with labral tears and were located in the anterior quadrant of the acetabulum [7]. In terms of location, the most common defect area is found in the anterosuperior acetabulum at the chondrolabral junction, usually due to FAI syndrome. In contrast, the most common defect area in the femur is generally found centrally in the head [2].

The treatment of articular cartilage injuries is challenging, especially in weight-bearing joints such as the hip. In addition, there is a concern regarding the safety and efficacy of surgical hip dislocation in managing femoral trauma [8]. However, hip arthroscopy has recently been gaining popularity as a safe, effective, and minimally invasive method of treating acute and chronic pathology [9]. Conservative treatment frequently yields unsatisfactory results because of the underlying injury to the femoral head cartilage and potential loose bodies that may compromise joint function, causing posttraumatic osteoarthritis to proceed rapidly [10]. Joint arthroplasty is the gold standard for reducing pain and restoring function, although in young, active patients, decreased implant longevity is a concern [11-16]. Although total hip arthroplasty (THA) or resurfacing may provide pain relief and return to activity, they might not be suitable options if the acetabulum remains intact. Additionally, young patients’ high activity levels could result in an early revision [17]. Total hip arthroplasty (THA) is indicated in advanced arthritis, whereas for focal chondral injury, various joint-preserving surgical procedures have been developed during the past few years. The majority of them are adaptations of well-known knee surgeries, such as debridement, microfracture, autologous chondrocyte implantation (ACI), matrix-induced autologous chondrocyte implantation (MACI), autologous matrix-induced chondrogenesis (AMIC), osteochondral autograft transplantation, osteochondral allograft transplantation, direct cartilage suture repair, fibrin adhesive, intra-articular bone marrow mesenchymal stem cell (BM-MSC) injection, artificial plug (TruFit®), and, more recently, partial resurfacing of the femoral head [2,3,16,18-22]. These alternative hip-preserving strategies are more useful in patients who are younger. It seems to be a good option to treat full-thickness chondral lesions with compromised subchondral bone due to hyaline cartilage and superior mechanical properties compared to fibrocartilage [19]. In mosaicplasty, chondral or osteochondral deficiencies in an affected joint are filled with autologous osteochondral cylindrical grafts from a non-weight-bearing articular surface. Recent studies revealed that mosaicplasty for femoral head osteochondral lesions showed promising results. Reviewing recent research on mosaicplasty and its effects on the hip joint, especially in the long term, was the aim of this study. We presumed that this surgical method would produce acceptable clinical results and a significant improvement in clinical scores in the short-, mid-, and long-term.

## Review

Literature review

Search Strategies and Inclusion Criteria

A systematic review was conducted on two databases (MEDLINE/PubMed and Scholar Google) using the keywords “Mosaicplasty,” “Hip osteochondral defect,” “Hip preserving surgery,” and “Hip osteochondral lesion” in the English language between January 1, 2000, and December 30, 2021. The exclusion criteria included age above 45 years, acetabular chondropathy, and femoral head osteonecrosis. Abstracts were screened by two reviewers (EA and PA) independently (population, intervention, comparison, outcomes, and study (PICOS) criteria).

Results

**Figure 1:**
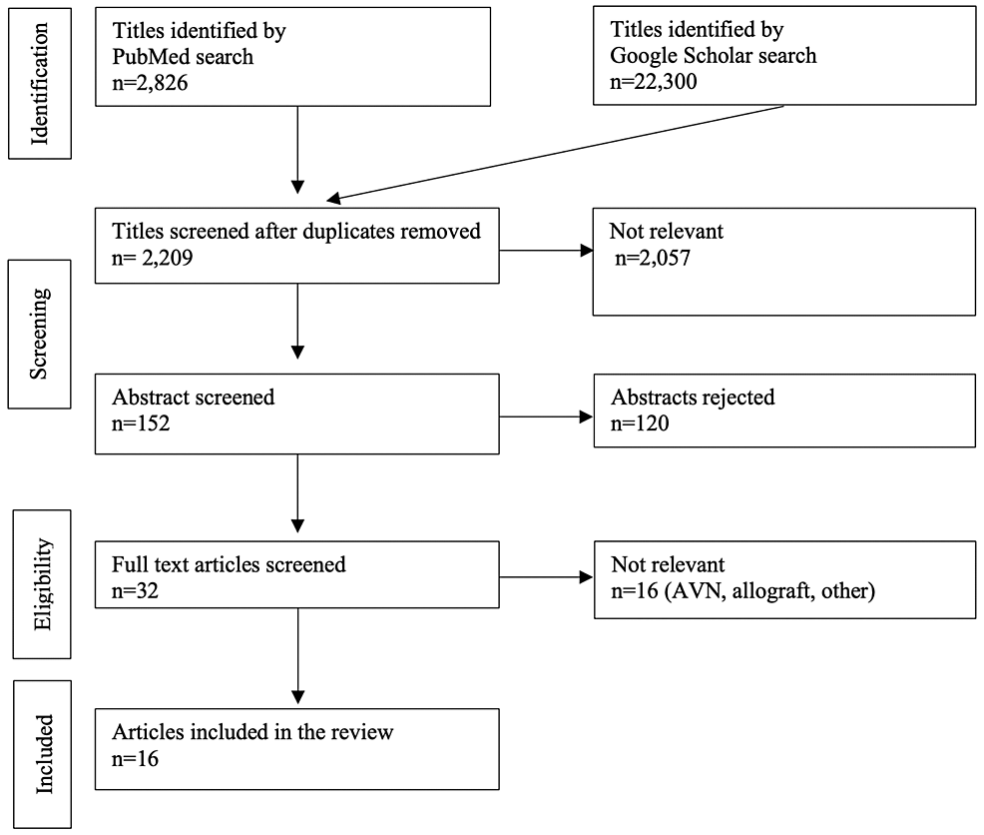
PRISMA flowchart of the study selection process PRISMA: Preferred Reporting Items for Systematic Reviews and Meta-Analysis, AVN: avascular necrosis

Data were extracted as follows: our review of the literature yielded 2,209 studies, of which 152 were eligible for abstract review and 32 for full-text review. Finally, 16 studies were found to be eligible for inclusion in our review (Figure 1).

Fifty-one (51) femoral head mosaicplasty procedures were published in 16 papers [23-38], which included short-, mid-, and long-term (one case) studies ranging from one case to 27 cases (Table 1). The etiology of the osteochondral femoral head defect included 21 trauma, 13 femoroacetabular impingement (FAI), four osteochondritis dissecans (OCD), one chondroblastoma, eight sequelae of Legg-Calvé-Perthes disease, and four epiphyseal dysplasia. There were 21 females and 30 males, with a mean age of 22.1 years, ranging from 15 to 44 years. Forty-seven (47) patients underwent open surgical procedures and four arthroscopic surgery (two retrograde and two antegrade mosaicplasty). The mean osteochondral defect size of the femoral head was 2.12 cm (range: 6 × 1 cm to 1 × 4 cm), and the mean number of autologous plunges was two (range: 1-8). The donor site of autologous graft in 10 cases was the ipsilateral knee and in 41 cases, the ipsilateral femoral head. The mean last follow-up was 37 months (range: 6-156 months). The results show good to excellent scores.

**Table 1:** Characteristics of the included studies M: male, F: female, PD: Perthes disease, FAI: femoroacetabular impingement, OCD: osteochondritis dissecans, AVN: avascular necrosis, HHS: Harris Hip Score, mHHS: modified Harris Hip Score

Study (year)/reference	Study design	Number of patients and gender (M/F)	Followed up (number)	Mean follow-up time (months)	Mean age (years)	Etiology	Location of the defect	Defect size	Number of plugs	Donor site	Treatment type (arthroscopic/open)	Complications	Results
Hart et al. (2003) [23]	Case report	1 M	1	6	28	Trauma (hip dislocation)	Femoral head	1.4 × 1.6 cm	4	Ipsilateral knee	Open	No	100 points (HHS)
Nam et al. (2010) [24]	Case report	2 M	2	36 (12 and 60)	18 (15 and 21)	Trauma (hip dislocation)	Femoral head	3 × 3 cm and 1 × 1 cm	3 and 1	Ipsilateral knee	Open	No	No complaints of pain
Girard et al. (2011) [25]	Case series	7 M, 3 F	10	29.2 (20-39)	18 (15-21)	PD (6 hips), epiphyseal dysplasia (4 hips)	Femoral head	1-3 × 3 cm	3-6	Ipsilateral femoral head	Open	1 sciatic nerve palsy that improved within three months	Postel Merle d'Aubigné score improved from 10.5 points (8-13) to 15.5 points (12-17)
Emre et al. (2012) [26]	Case report	1 M	1	6	22	PD	Femoral head	1.6 × 1.8 cm	3	Ipsilateral knee	Open	No	96 points (HHS)
Philippon et al. (2012) [27]	Case report	1 F	1	25	15	Trauma	Femoral head	0.6 × 1 cm	1	Ipsilateral femoral head	Arthroscopic	Iliotibial snapping symptoms	85 points (HHS)
Krych et al. (2012) [28]	Case report	1 M, 1 F	2	49 (50 and 48)	22 (15 and 29)	Trauma	Femoral head	2 × 0.5-0.8 cm and 1 × 2 cm	3 and 1	Ipsilateral knee	Open	No	96 and 100 points (mHHS)
Güngör et al. (2015) [29]	Case report	2 F	2	13 (12 and 14)	22.5 (22 and 23)	FAI	Femoral head	2.7 × 1 cm and 3.6 × 1 cm	3 and 4	Ipsilateral knee	Open	No	85 and 93 points (HHS)
Anthonissen et al. (2015) [30]	Case report	1 M	1	28	20	Trauma (hip dislocation)	Femoral head	2 × 2.5 cm	4	Ipsilateral knee	Open	Pain over the screw heads	84 points (HHS)
Zelken et al. (2016) [31]	Case report	1 M	1	156	21	Trauma (hip dislocation)	Femoral head	1 cm	1	Ipsilateral femoral head	Open	Pulmonary embolism, lateral hip pain	100 points (HHS)
Kocadal et al. (2017) [32]	Case report	1 M	1	26	27	Trauma	Femoral head	1 cm	1	Ipsilateral knee	Arthroscopic (retrograde mosaicplasty)	No	96 points (HHS)
Uchida et al. (2017) [33]	Report article	1 M, 1 F	2	25 (14 and 36)	39 (18 and 40)	OCD	Femoral head	0.85 cm and 1 cm	1 and 1	Ipsilateral knee	Arthroscopic (one antegrade and one retrograde mosaicplasty)	No	One HHS improved from 72.5 to 87.5 points, the other from 88.7 to 100 points
Johnson et al. (2017) [34]	Research article	5 F	5	52 (30, 52, 62, and 64)	21.7 (16, 21, 25, and 25)	1 AVN and 4 trauma	Femoral head	1-4 cm	1-3	Ipsilateral femoral head	Open	Hardware removal (20%)	HHS improved to 95-100 points (no AVN included)
Verma et al. (2021) [35]	Case report	1 F	1	24	17	Chondroblastoma	Femoral head	2.6 × 1.8 cm	3	Ipsilateral knee	Open	No	Pain-free
Viamont-Guerra et al. (2019) [36]	Case series	17 M, 10 F	22	34.1 (12-90.2)	29 ± 7 (19-44)	11 FAI, 7 trauma, 4 AVN, 2 osteochondritis	Femoral head	1.6 × 2.5 cm	1-8	Ipsilateral femoral head	Open	1 THA (4%)	Their mHHS improved from 56.3 ± 12.6 to 88.4 ± 9.9 (2% disappointed)
Palazón-Quevedo et al. (2021) [37]	Case report	1 M	1	52	15	Perthes disease	Femoral head	No report	3	Ipsilateral femoral head	Open	No	85.85% (HHS)
Coulomb et al. (2021) [38]	Case report	1 M	1	12	16	Trauma	Femoral head	3 × 2 cm	3	Ipsilateral knee	Open	Knee pain and stiffness	93 points (HHS)

Statistical Analysis

Statistical analysis was determined by two objectives. The first objective was to assess differences in HHS distributions among the individual causes (i.e., trauma, PD, FAI, dysplasia, and OCD). The second objective was to pool the individual HHS mean estimates of the aforementioned causes and conclude with a single (pooled) outcome using a set of meta-analysis methods for averages. To accomplish the first objective, a one-way analysis of variance (ANOVA) was performed, with the use of summary statistics (total count, mean value, and standard deviation value). Moreover, post hoc pairwise comparisons were done using Tukey’s honestly significant difference (HSD) test to conclude to bivariate differences [39].

Meta-analysis for averages (i.e., pooling the effect size and the HHS mean) was done using the inverse variance method. Tau2 was computed using the Sidik-Jonkman (SJ) estimator, while its 95% confidence intervals (CI) using the Q-profile method. The Hartung-Knapp adjustment for the random effects model was also utilized. Overall, the random effects model was used, and means were considered untransformed (raw). Heterogeneity was quantified with the indices I2 and tau2, as well as with Cochran’s Q test. The outcome visualization was performed with a summary forest plot. Lastly, a meta-regression process that combined the SJ estimator and the “knha” significance test was executed to identify potential predictors that drove the heterogeneity of the study (subjects’ age, follow-up time, and surgery type) [40].

All statistical tests were considered as two-sided, and statistical significance was taken when p < 0.05. Implementation was held with R, the language for statistical computing, along with the RStudio IDE, both of which are well-known open-source products. Pooling was implemented using the library “meta” [41].

Results

**Figure 2:**
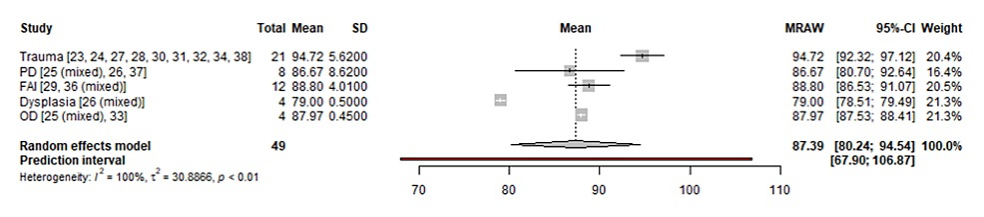
Meta-analysis process PD: Perthes disease, FAI: femoroacetabular impingement, OD: osteochondritis dissecans, SD: standard deviation, CI: confidence interval

A comparison among the HHS distributions for different causes showed statistical significance (p < 0.001) overall. More specifically, the pairs that seem to differ considerably are trauma versus dysplasia (p < 0.001), trauma versus PD (p = 0.008), FAI versus dysplasia (p = 0.027), and trauma versus FAI (p = 0.036). One may observe visually the individual distributions in Figure 2 (forest plot). Moreover, one may observe the executed pooling outcome, where the aggregated average of HHS is 87.39 (95% CI: 80.24-94.54). Heterogeneity was quantified by tau2 = 30.89, I2 = 99.5%, and Cochran’s Q test p < 0.001. The meta-regression process that was finally executed revealed that no potential predictor was eventually affecting the outcome, including the average age of the patients, the average follow-up time, and the fraction of open surgeries (defined as the ratio of open to the sum of open and arthroscopic surgeries) (Figure 2).

Discussion

Chondral pathology has been categorized using several different classification systems [19,20]. Sampson proposed a classification system specific to cartilage lesions of the femoral head and acetabulum. Based on this classification, he recommended treatment protocols [42]. Regarding the treatment of femoral cartilage lesions, algorithms have been suggested [2,3,20,43].

Wilson and Jacobs reported the first osteochondral autograft transplantation in 1952 using a patellar graft for a lateral tibial plateau fracture [44]. However, mosaicplasty for osteochondral lesions was described for the first time by Hangody in 1997, and since then, its popularity has risen [45]. The long-term survival of the transplanted chondrocytes and osteocytes has been demonstrated by histological studies [46-49]. The talus, tibial plateau, patella, humeral capitellum, and femoral head are among the various articular surfaces to which mosaicplasty methods have been applied as a result of their effectiveness in the knee. Hangody and Füles reviewed 831 patients who underwent mosaicplasties over 10 years at their institution and found good to excellent results in 92% of patients with femoral condylar implantations, 87% with tibial resurfacing, 79% with patellar and/or trochlear mosaicplasties, and 94% with talar procedures [50]. In their 17-year prospective multicenter study of 303 knee, 39 talar, and 12 elbow autograft transplantations, Hangody et al. reported the findings. A minor deterioration in their performance was observed during the 10-year follow-up period, although follow-up data still showed good to exceptional results [51]. Hangody and Füles reported that osteochondral transplantation was performed on six femoral heads. However, the specifics of the surgical process and the clinical outcome were not covered [50].

Gole et al. found that an osteochondral graft’s load-bearing had a beneficial impact on cell viability, indicating that grafts positioned in weight-bearing areas will function better than those positioned in other areas [52]. The better results attained by younger patients suggest that age may have an impact on the clinical outcomes of mosaicplasty treatments [10,53]. After evaluating the outcomes of 831 cartilage joints treated with mosaicplasty, Bartha et al. concluded that results are less remarkable after 45 years of age and that 50 may be the maximum age limit [54]. The optimal defect coverage ranges from 1 to 4 cm^2^, depending on the availability of donor sites and other technical factors [54,55]. It has been shown that expanding the criteria to include larger knee lesions (8-9 cm^2^) results in an increased rate of donor site morbidity [50,54]. Although the majority of authors employed the ipsilateral knee’s lateral femoral condyle for transplant harvesting, Girard et al. hypothesized that the femoral head’s non-weight-bearing portion might be advantageous [25]. They cited Mardones et al. who reported that excision of up to 30% of the anterolateral quadrant of the femoral head did not appreciably affect the proximal part of the femoral head’s ability to bear weight [56]. Smaller lesions may be suitable for the non-weight-bearing part of the femoral head, particularly when only one cylinder is required. However, grafts from the lateral femoral condyle should be used for bigger lesions [30].

Girard et al. and Viamont-Guerra et al. have published case series of mosaicplasty of the femoral head [25,36]. Girard et al. reported 10 patients with osteochondral lesions of the femoral head who underwent mosaicplasty through trochanteric flap hip dislocation. Sequelae of Legg-Calvé-Perthes disease (six hips), spondylo-epiphyseal dysplasia (three hips), and epiphyseal dysplasia (one hip) were the causes of the osteochondral femoral head defect. They used bone grafts from the non-weight-bearing surface of the ipsilateral femoral head with plugs ranging from 6 to 10 mm. It was suggested that non-weight-bearing for six weeks be followed by gradually increasing weight-bearing as tolerated. The mean follow-up was 29.2 (20-39) months. The Postel Merle d’Aubigné score improved from the preoperative period to the latest follow-up, from 10.5 (8-13) points to 15.5 (12-17) points, and the Harris Hip Score increased from 52.8 (35-74) points to 79.5 (65-93) points, respectively [25]. Viamont-Guerra et al. reported a series of 27 mosaicplasties. The osteochondral lesion of the femoral head was 1.6 ± 0.7 (range: 0.8-4.0) cm^2^ in patients aged 28.7 ± 7.4 (range: 19-44) years. The etiology of the osteochondral defect was FAI, posttraumatic, osteochondritis, and avascular necrosis (the four AVN are excluded from our study). In all patients, osteochondral plugs were taken from the non-weight-bearing surface of the femoral head through a minimally invasive anterior (Hueter) surgical approach. The average diameter of the autografted plugs was 8.5 ± 1.3 (range: 6-10) mm. Toe-touch weight-bearing on the operated limb was allowed for the first 4-6 weeks and then progressed to total weight-bearing as tolerated. At the final follow-up, one patient had been revised to total hip arthroplasty (THA) due to persistent hip pain and the development of degenerative coxarthrosis. The average follow-up of the remaining 22 patients was 39 ± 23 (12-90) months. Their mHHS improved from 56 ± 13 to 88 ± 10, and their Western Ontario and McMaster Universities Osteoarthritis Index (WOMAC) improved from 45 ± 17 to 81 ± 13. Twenty (91%) patients were very satisfied or satisfied with the surgery [36]. They found that harvesting plugs from the ipsilateral femur via a minimally invasive anterior approach provides satisfactory outcomes and functional improvements. However, they advised that it should be considered for up to 2 cm^2^ (diameter: 16 mm) lesions of the femoral head.

Studies have shown that the long-term clinical outcome after mosaicplasty varies greatly depending on age, gender, and the size of the lesion [31,47,51,54,57-63].

Zelken presented the first long-term follow-up (13 years) as a patient and surgeon after a posttraumatic type II Pipkin fracture. After the fracture of femoral head fixation, the osteochondral defect of the femoral head was filed with a plug of 10 mm harvested from the non-weight-bearing anterior inferior surface of the ipsilateral femoral head. Mobilized touchdown weight-bearing for the first six weeks postoperatively was allowed, followed by gradually increasing weight-bearing. Thirteen years later, he reported being pain-free with a Harris Hip Score of 100 [31]. Another case with an eight-year follow-up after a successful femoral head mosaicplasty was reported by Kılıçoğlu et al. [57]. However, it was caused by avascular necrosis, which is not one of our criteria [57].

This is the first systematic review of mosaicplasty of the femoral head to our knowledge. This study shows satisfactory short- and medium-term results and promising in the long term. The comparison among the HHS distributions for different causes showed statistical significance (p < 0.001) overall. More specifically, the pairs that seem to differ considerably are trauma versus dysplasia (p < 0.001), trauma versus PD (p = 0.008), FAI versus dysplasia (p = 0.027), and trauma versus FAI (p = 0.036).

The present study has a number of limitations that should be mentioned. The first limitation is the heterogeneity in surgical techniques, imaging modalities, and groups of patients. The second is that the number of patients remains insufficient overall and in each separate group. The third is the time of follow-up, which is almost short- and medium-term.

## Conclusions

The indication for cartilage procedures continues to evolve for the knee, and a similar strategy may be adopted for the hip joint. Due to limited evidence and a lack of experience, mosaicplasty treatment of these lesions remains challenging, especially in young patients. This study shows that open and arthroscopic management using the knee and femoral head as donor sites yielded good to excellent short- to mid-term outcomes. For osteochondral lesions of the femoral head, mosaicplasty may be a new alternative treatment option, although this needs to be proven with longer follow-ups and in a larger sample of patients. Long-term studies and postoperative MRIs would help determine the procedure’s success.
